# Supplementary material for: Built Environment and Health Behaviors: Deconstructing the Black Box of Interactions—A Review of Reviews
Source: Int J Environ Res Public Health. 2019 Apr 24;16(8):1454. doi: 10.3390/ijerph16081454 (PMC6517917; doi:10.3390/ijerph16081454)
Supplement: Supplementary file 1 [file ijerph-16-01454-s001.zip › ijerph-444662 Supplementary Materials - Final/Additional File 1 - Search strategy.pdf]

Supplementary Table S1 – Subject headings and text word search

Databases	Keywords lists	Mesh-terms	Emtree-terms	Text words
PubMed, Web of Science, Cochrane Library, Embase	Diet	Diet; Diet, Food, and Nutrition; Healthy diet; Feeding behavior; Eating	Diet; Nutrition; Healthy diet; Feeding behavior; Eating; Food choice; Unhealthy diet; Unhealthy food	Food decisions; Food choices; Unhealthy foods; Unhealthy diets; Eat
	Physical activity	Exercise; Sports; Running; Walking; Bicycling; Sedentary lifestyle	Physical activity; Exercise; Sport; Running; Walking; Biking; Cycling; Commuting; Active commuting; Sedentary life style; Sedentary behavior; Physical inactivity; Inactivity	Physical activity; Sport; Biking; Bike; Bicycle; Bicycling; Cycling; Walk*; Strolling; School recess; Active recreation; Active living; Active transportation; Active commuting; Active travel; Commuting; Transportation modes; Transportation choices; Sedentary behaviors; Physical inactivity; Inactivity
	Built environment	Environment design; City planning; Architecture; Spatial behavior	Built environment; Physical environment; Environmental planning; City planning; Architecture; Spatial behavior	Built environment; Physical environment; Neighborhood environment; Urban planning; Urban design; Environment design; Environmental planning; Streetscape; Foodscape; Urban form

Supplementary Table S2 – Search strategy for PubMed

**PubMed**<br>6 February 2018
**<u>Built environment and physical activity terms</u>**<br><br>("exercise"[MeSH Terms] OR "sports"[MeSH Terms] OR "running"[MeSH Terms] OR "walking"[MeSH Terms] OR "bicycling"[MeSH Terms] OR "sedentary lifestyle"[MeSH Terms] OR "physical activity"[All Fields] OR "sports"[MeSH Terms] OR "sports"[All Fields] OR "sport"[All Fields] OR biking[All Fields] OR "bicycling"[MeSH Terms] OR "bicycling"[All Fields] OR "bike"[All Fields] OR bicycle[All Fields] OR "bicycling"[MeSH Terms] OR "bicycling"[All Fields] OR cycling[All Fields] OR "walking"[MeSH Terms] OR "walking"[All Fields] OR "walk"[All Fields] OR strolling[All Fields] OR "school recess"[All Fields] OR "active recreation"[All Fields] OR "active living"[All Fields] OR "active transportation"[All Fields] OR "active commuting"[All Fields] OR "active travel"[All Fields] OR "transportation"[MeSH Terms] OR "transportation"[All Fields] OR "commuting"[All Fields] OR "transportation modes"[All Fields] OR "transportation choices"[All Fields] OR "sedentary behaviors"[All Fields] OR "physical inactivity"[All Fields] OR inactivity[All Fields]) AND ("environment design"[MeSH Terms] OR "city planning"[MeSH Terms] OR "architecture"[MeSH Terms] OR "spatial behavior"[MeSH Terms] OR "built environment"[All Fields] OR "physical environment"[All Fields] OR "neighborhood environment"[All Fields] OR "urban planning"[All Fields] OR "urban design"[All Fields] OR "environment design"[All Fields] OR "environmental planning"[All Fields] OR streetscape[All Fields] OR foodscape[All Fields] OR "urban form"[All Fields]) AND (Meta-Analysis[ptyp] OR systematic[sb]) AND ("2007/01/01"[PDAT] : "2017/12/31"[PDAT]) AND "humans"[MeSH Terms] AND English[lang]
**<u>Built environment and diet terms</u>**<br><br>(("Diet"[Mesh] OR "Diet, Food, and Nutrition"[Mesh] OR "Healthy Diet"[Mesh] OR "Feeding Behavior"[Mesh] OR "Eating"[Mesh] OR "food decisions"[All Fields] OR "food choices"[All Fields] OR "Unhealthy foods"[All Fields] OR "unhealthy diets"[All Fields] OR eat[All Fields]) AND ("environment design"[MeSH Terms] OR "city planning"[MeSH Terms] OR "architecture"[MeSH Terms] OR "spatial behavior"[MeSH Terms] OR "built environment"[All Fields] OR "physical environment"[All Fields] OR "neighborhood environment"[All Fields] OR "urban planning"[All Fields] OR "urban design"[All Fields] OR "environment design"[All Fields] OR "environmental planning"[All Fields] OR streetscape[All Fields] OR foodscape[All Fields] OR "urban form"[All Fields])) AND ((Meta-Analysis[ptyp] OR systematic[sb]) AND ("2007/01/01"[PDAT] : "2017/12/31"[PDAT]) AND "humans"[MeSH Terms] AND English[lang])
**Total retrieved: 206**

Supplementary Table S3 – Search strategy for Web of Science

**Web of Science**<br>6 February 2018
**<u>Built environment and physical activity terms</u>**<br><br>((TS=("exercise" OR "physical activity" OR "sport" OR "running" OR "walk*" OR walking OR "Biking" OR "bike" OR "bicycle" OR "bicycling" OR "cycling" OR "commuting" OR "active commuting" OR "active transportation" OR "sedentary life style" OR "active living" OR "active travel" OR "sedentary behaviors" OR "physical inactivity" OR "inactivity" OR "strolling" OR "school recess" OR "active recreation" OR "transportation modes" OR "transportation choices")) AND (TS=("environment design" OR "built environment" OR "physical environment" OR "environmental planning" OR "city planning" OR "spatial behavior" OR "neighborhood environment" OR "urban planning" OR "urban design" OR "streetscape" OR "foodscape"))) *AND* **LANGUAGE:** (English) *AND* **DOCUMENT TYPES:** (Review)<br>*Indexes=SCI-EXPANDED, SSCI, A&HCI, CPCI-S, CPCI-SSH, ESCI Timespan=2007-2017*
**<u>Built environment and diet terms</u>**<br><br>((TS= (("diet" OR "food" OR "nutrition" OR "healthy diet" OR "feeding behavior" OR "eating" OR "food choices" OR "food decisions" OR "unhealthy foods" OR "unhealthy diets" OR "eat") AND ("environment design" OR "built environment" OR "physical environment" OR "environmental planning" OR "city planning" OR "spatial behavior" OR "neighborhood environment" OR "urban planning" OR "urban design" OR "streetscape" OR "foodscape")))) *AND* **LANGUAGE:** (English) *AND* **DOCUMENT TYPES:** (Review)<br>*Indexes=SCI-EXPANDED, SSCI, A&HCI, CPCI-S, CPCI-SSH, ESCI Timespan=2007-2017*
**Total retrieved: 377**

Supplementary Table S4 – Search strategy for Cochrane Library

**Cochrane Library**<br>7 February 2018
**<u>Built environment and physical activity terms</u>**<br><br>#1 MeSH descriptor: [Environment Design] explode all trees<br>#2 MeSH descriptor: [City Planning] explode all trees<br>#3 MeSH descriptor: [Architecture] explode all trees<br>#4 MeSH descriptor: [Spatial Behavior] explode all trees<br>#5 "built environment"<br>#6 "physical environment"<br>#7 "neighborhood environment"<br>#8 "urban planning"<br>#9 "urban design"<br>#10 "environment design"<br>#11 "environmental planning"<br>#12 streetscape<br>#13 foodscape<br>#14 "urban form"<br>#15 #1 or #2 or #3 or #4 or #5 or #6 or #7 or #8 or #9 or #10 or #11 or #12 or #13 or #14<br>#16 MeSH descriptor: [Exercise] explode all trees<br>#17 MeSH descriptor: [Sports] explode all trees<br>#18 MeSH descriptor: [Running] explode all trees<br>#19 MeSH descriptor: [Walking] explode all trees<br>#20 MeSH descriptor: [Bicycling] explode all trees<br>#21 MeSH descriptor: [Sedentary Lifestyle] explode all trees<br>#22 "physical activity"<br>#23 sport<br>#24 biking<br>#25 bike<br>#26 bicycle<br>#27 bicycling<br>#28 cycling<br>#29 walk*<br>#30 strolling<br>#31 "school recess"<br>#32 "active recreation"<br>#33 "active living"<br>#34 "active transportation"<br>#35 "active commuting"<br>#36 "active travel"<br>#37 commuting<br>#38 "transportation modes"<br>#39 "transportation choices"<br>#40 "sedentary behaviors"<br>#41 "physical inactivity"<br>#42 inactivity<br>#43 #16 or #17 or #18 or #19 or #20 or #21 or #22 or #23 or #24 or #25 or #26 or #27 or #28 or #29 or #30 or #31 or #32 or #33 or #34 or #35 or #36 or #37 or #38 or #39 or #40 or #41 or #42

#44	#15 and #43 Publication Year from 2007 to 2017, in Other Reviews

<u>Built environment and diet terms</u>

#1	MeSH descriptor: [Diet] explode all trees
#2	MeSH descriptor: [Diet, Food, and Nutrition] explode all trees
#3	MeSH descriptor: [Healthy Diet] explode all trees
#4	MeSH descriptor: [Feeding Behavior] explode all trees
#5	MeSH descriptor: [Eating] explode all trees
#6	"food decisions"
#7	"food choices"
#8	"unhealthy foods"
#9	"unhealthy diets"
#10	eat
#11	#1 or #2 or #3 or #4 or #5 or #6 or #7 or #8 or #9 or #10
#12	MeSH descriptor: [Environment Design] explode all trees
#13	MeSH descriptor: [City Planning] explode all trees
#14	MeSH descriptor: [Architecture] explode all trees
#15	MeSH descriptor: [Spatial Behavior] explode all trees
#16	"built environment"
#17	"physical environment"
#18	"neighborhood environment"
#19	"urban planning"
#20	"urban design"
#21	"environment design"
#22	"environmental planning"
#23	streetscape
#24	foodscape
#25	"urban form"
#26	#12 or #13 or #14 or #15 or #16 or #17 or #18 or #19 or #20 or #21 or #22 or #23 or #24 or #25
#27	#11 and #26 Publication Year from 2007 to 2017, in Other Reviews

Total retrieved: 37

Supplementary Table S5 – Search strategy for Embase

**Embase**<br>7 February 2018
**Built environment and physical activity terms**<br><br>('exercise'/exp OR 'exercise' OR 'sport' OR 'running'/exp OR 'running' OR 'walking'/exp OR 'walking' OR 'biking' OR 'commuting' OR 'sedentary lifestyle'/exp OR 'sedentary lifestyle' OR 'sedentary behavior'/exp OR 'sedentary behavior' OR 'inactivity' OR 'physical activity'/exp OR 'physical activity' OR 'sport'/exp OR sport OR 'biking'/exp OR biking OR bike OR 'bicycle'/exp OR bicycle OR 'bicycling'/exp OR bicycling OR 'cycling'/exp OR 'cycling' OR walk* OR strolling OR 'school recess' OR 'active recreation' OR 'active living' OR 'active transportation' OR 'active commuting'/exp OR 'active commuting' OR 'active travel' OR 'commuting'/exp OR commuting OR 'transportation modes' OR 'transportation choices' OR 'sedentary behaviors' OR 'physical inactivity'/exp OR 'physical inactivity' OR 'inactivity'/exp OR inactivity) AND ('city planning'/exp OR 'city planning' OR 'architecture'/exp OR 'architecture' OR 'spatial behavior'/exp OR 'spatial behavior' OR 'built environment'/exp OR 'built environment' OR 'physical environment'/exp OR 'physical environment' OR 'neighborhood environment' OR 'urban planning'/exp OR 'urban planning' OR 'urban design' OR 'environment design'/exp OR 'environment design' OR 'environmental planning'/exp OR 'environmental planning' OR streetscape OR foodscape OR 'urban form') AND ([cochrane review]/lim OR [systematic review]/lim OR [meta analysis]/lim) AND [english]/lim AND [humans]/lim AND ([embase]/lim OR [medline]/lim) AND [2007-2017]/py
**Built environment and diet terms**<br><br>('diet'/exp OR 'diet' OR 'nutrition'/exp OR 'nutrition' OR 'healthy diet'/exp OR 'healthy diet' OR 'feeding behavior'/exp OR 'feeding behavior' OR 'eating'/exp OR 'eating' OR 'food choice'/exp OR 'food choice' OR 'unhealthy diet'/exp OR 'unhealthy diet' OR 'unhealthy food'/exp OR 'unhealthy food' OR 'food decisions' OR 'food choices' OR 'unhealthy diets' OR 'unhealthy foods' OR 'eat') AND ('city planning'/exp OR 'city planning' OR 'architecture'/exp OR 'architecture' OR 'spatial behavior'/exp OR 'spatial behavior' OR 'built environment'/exp OR 'built environment' OR 'physical environment'/exp OR 'physical environment' OR 'neighborhood environment' OR 'urban planning'/exp OR 'urban planning' OR 'urban design' OR 'environment design'/exp OR 'environment design' OR 'environmental planning'/exp OR 'environmental planning' OR streetscape OR foodscape OR 'urban form') AND ([cochrane review]/lim OR [systematic review]/lim OR [meta analysis]/lim) AND [english]/lim AND [humans]/lim AND ([embase]/lim OR [medline]/lim) AND [2007-2017]/py
**Total retrieved: 212**

Supplementary Table S6 – Search strategy for the updated review (2018-2019)

The review was updated in March 2019. The same search strategies were run in the 4 online databases in order to retrieved records published between 1 January 2018 and 18 March 2019.

PubMed	10
Web of Science	87
Cochrane Library	0
Embase	71
**Total retrieved**	**168**
