# Supplementary material for: Built Environment and Health Behaviors: Deconstructing the Black Box of Interactions—A Review of Reviews
Source: Int J Environ Res Public Health. 2019 Apr 24;16(8):1454. doi: 10.3390/ijerph16081454 (PMC6517917; doi:10.3390/ijerph16081454)
Supplement: Supplementary file 1 [file ijerph-16-01454-s001.zip › ijerph-444662 Supplementary Materials - Final/Additional File 2 - Excluded records.pdf]

Supplementary Table S7 – Excluded records with reasons for exclusion (n=61)

Records	Reason for exclusion
Afshin, A., Penalvo, J., Del Gobbo, L., Kashaf, M., Micha, R., Morrish, K., … Mozaffarian, D. (2015). CVD Prevention Through Policy: a Review of Mass Media, Food/Menu Labeling, Taxation/Subsidies, Built Environment, School Procurement, Worksite Wellness, and Marketing Standards to Improve Diet. *Current Cardiology Reports*, *17*(11), 98.	Not a systematic search
Anaby, D., Hand, C., Bradley, L., DiRezze, B., Forhan, M., DiGiacomo, A., & Law, M. (2013). The effect of the environment on participation of children and youth with disabilities: a scoping review. *Disability and Rehabilitation*, *35*(19), 1589–1598.	Focus on participation as the main outcome and not on diet or PA
Anderson, W. P.; Steyn, N. P. (2009). Interventions on diet and physical activity: What works summary report. *Geneva: Wold Health Organization*, 48.	The built environment is not specifically examined
Beard, J. R., & Petitot, C. (2011). Ageing and urbanization: Can cities be designed to foster active ageing? *Public Health Reviews*.	No specific focus on PA related outcomes in relationship to the built environment
Biddle, S. J. H., Atkin, A. J., Cavill, N., & Foster, C. (2011). Correlates of physical activity in youth: a review of quantitative systematic reviews. *International Review of Sport and Exercise Psychology*, *4*(1), 25–49.	No specific focus on the built environment
Bird, E.L., Ige, J.O., Pilkington, P., Pinto, A., Petrokofsky, C., Burgess-Allen, J., (2018). Built and natural environment planning principles for promoting health: an umbrella review. BMC Public Health.18.	Review of reviews describing papers already selected
Bodde, A. E., & Seo, D.-C. (2009). A review of social and environmental barriers to physical activity for adults with intellectual disabilities. *Disability and Health Journal*, *2*(2), 57–66.	No specific focus on the built environment which is not being examined in detail
Broome, K., McKenna, K., Fleming, J., & Worrall, L. (2009). Bus use and older people: a literature review applying the Person-Environment-Occupation model in macro practice. *Scandinavian Journal of Occupational Therapy*, *16*(1), 3–12.	Not a systematic search
Brown, V., Moodie, M., & Carter, R. (2017). Evidence for associations between traffic calming and safety and active transport or obesity: A scoping review. *Journal of Transport & Health*, *7*(A), 23–37.	No direct relationship examined between specific built environment features and PA outcomes
Brownson, R. C., Hoehner, C. M., Day, K., Forsyth, A., & Sallis, J. F. (2009). Measuring the Built Environment for Physical Activity. State of the Science. *American Journal of Preventive Medicine*, *36*(4 SUPPL.), S99–S123.e12.	Not a systematic search
Bult, M. K., Verschuren, O., Jongmans, M. J., Lindeman, E., & Ketelaar, M. (2011). What influences participation in leisure activities of children and youth with physical disabilities? A systematic review. *Research in Developmental Disabilities*, *32*(5), 1521–1529.	Do not focus on the relationship between the built environment and PA outcomes

Casey, R., Oppert, J. M., Weber, C., Charreire, H., Salze, P., Badariotti, D., … Simon, C. (2014). Determinants of childhood obesity: What can we learn from built environment studies? *Food Quality and Preference*, *31*(1), 164–172.	Focus on weight as the main outcome
Chillón, P., Evenson, K. R., Vaughn, A., & Ward, D. S. (2011). A systematic review of interventions for promoting active transportation to school. *The International Journal of Behavioral Nutrition and Physical Activity*, *8*(1), 10.	No focus on the relationship between the built environment and PA
Cui, J., Loo, B. P. Y., & Lin, D. (2017). Travel behaviour and mobility needs of older adults in an ageing and car-dependent society. *International Journal of Urban Sciences*, *21*(2), 109–128.	Not a systematic search
D'Haese, S.; De Bourdeaudhuij, I.; Deforche, B.; Hinckson, E. . C., & G. (2012). A systematic review of the relationship between the built environment and different domains of physical activity in children. *Journal of Science and Medicine in Sport*, *15*, S253.	This article could not be accessed
Davison, K. K., Werder, J. L., Lawson, C. T. (2008). Children's active commuting to school: current knowledge and future directions. *Preventing Chronic Disease*, *5*(3), A100.	Not a systematic search
De Sa, J., & Lock, K. (2008). Will European agricultural policy for school fruit and vegetables improve public health? A review of school fruit and vegetable programmes. *European Journal of Public Health*, *18*(6), 558–568.	No focus on the built environment as a disaggregated factor
De Vet, E., de Ridder, D. T. D., & de Wit, J. B. F. (2011). Environmental correlates of physical activity and dietary behaviours among young people: A systematic review of reviews. *Obesity Reviews*, *12*(501).	Review of reviews describing papers already selected
Ding, D., & Gebel, K. (2012). Built environment, physical activity, and obesity: What have we learned from reviewing the literature? *Health and Place*, *18*(1), 100–105.	Focus on the methodology of review papers
Ferdinand, A., O; Sen, B.; Rahurkar, S.; Engler, S.; Menachemi, N. (2012). The relationship between built environments and physical activity: a systematic review. *American Journal of Public Health*, *102*(10), e7–e13.	Focus on the methodology of primary studies
Ferreira, I., Van Der Horst, K., Wendel-Vos, W., Kremers, S., Van Lenthe, F. J., & Brug, J. (2007). Environmental correlates of physical activity in youth - A review and update. *Obesity Reviews*.	No focus on the built environment
Fleischhacker, S. E., Evenson, K. R., Rodriguez, D. A., & Ammerman, A. S. (2011). A systematic review of fast food access studies. *Obesity Reviews*, *12*(501), e460–e471. https://doi.org/10.1111/j.1467-789X.2010.00715.x	No focus on the relationship of interest
Foster, S., & Giles-Corti, B. (2008). The built environment, neighborhood crime and constrained physical activity: An exploration of inconsistent findings. *Preventive Medicine*, *47*(3), 241–251.	Global measures mixing built environment, SES and individual factors
Gim, T. H. T. (2013). The relationships between land use measures and travel behavior: A meta-analytic approach. *Transportation Planning and Technology*, *36*(5), 413–434.	Travel understood as a broad category encompassing both motorized and non-motorized travels. No focus on active travel
Giskes, K., Kamphuis, C. B. M., Van Lenthe, F. J., Kremers, S., Droomers, M., & Brug, J. (2007). A systematic review of associations between environmental factors, energy and fat intakes among adults: Is there evidence for environments that encourage obesogenic dietary intakes? *Public Health Nutrition*, *10*(10), 1005–1017.	No focus on the built environment

Gomez, L. F., Sarmiento, R., Fernanda Ordonez, M., Felipe Pardo, C., de Sa, T. H., Mallarino, C. H., … Quistberg, D. A. (2015). Urban environment interventions linked to the promotion of physical activity: A mixed methods study applied to the urban context of Latin America. *Social Science & Medicine*, *131*, 18–30.	Review of reviews describing papers already selected
Gray, J. A., Zimmerman, J. L., & Rimmer, J. H. (2012). Built environment instruments for walkability, bikeability, and recreation: Disability and universal design relevant? *Disability and Health Journal*, *5*(2), 87–101.	No examination of the relationship between the built environment features measured and PA levels
Gustafson, A., Hankins, S., & Jilcott, S. (2012). Measures of the Consumer Food Store Environment: A Systematic Review of the Evidence 2000-2011. *Journal of Community Health*, *37*(4), 897–911. https://doi.org/10.1007/s10900-011-9524-x	No focus on the relationship of interest
Hansen, A. Y., Umstattd Meyer, M. R., Lenardson, J. D., & Hartley, D. (2015). Built Environments and Active Living in Rural and Remote Areas: a Review of the Literature. *Current Obesity Reports*, *4*(4), 484–493.	Not a systematic search
Haselwandter, E. M., Corcoran, M. P., Folta, S. C., Hyatt, R., Fenton, M., & Nelson, M. E. (2015). The Built Environment, Physical Activity, and Aging in the United States: A State of the Science Review. *Journal of Aging and Physical Activity*, *23*(2), 323–329.	This article could not be accessed
Hinkley, T., Crawford, D., Salmon, J., Okely, A. D., & Hesketh, K. (2008). Preschool Children and Physical Activity. A Review of Correlates. *American Journal of Preventive Medicine*.	No focus on the built environment
Jaarsma, E. A., Dijkstra, P. U., Geertzen, J. H. B., & Dekker, R. (2014). Barriers to and facilitators of sports participation for people with physical disabilities: A systematic review. *Adapted Physical Activity Quarterly*, *31*, 1–11.	No focus on the built environment
Kaczynski, A. T., & Henderson, K. A. (2008). Parks and Recreation Settings and Active Living: A Review of Associations With Physical Activity Function and Intensity. *Journal of Physical Activity & Health*, *5*(4), 619–632.	This article could not be accessed
Kerr, J., Rosenberg, D., & Frank, L. (2012). The Role of the Built Environment in Healthy Aging: Community Design, Physical Activity, and Health among Older Adults. *Journal of Planning Literature*, *27*(1), 43–60.	Not a systematic search
Kirk, S. F. L., Penney, T. L., & McHugh, T.-L. F. (2010). Characterizing the obesogenic environment: the state of the evidence with directions for future research. *Obesity Reviews*, *11*(2), 109–117.	PA is one of the outcomes but there is no particular focus on its examination in relation to the built environment
Koohsari, M. J., Sugiyama, T., Sahlqvist, S., Mavoa, S., Hadgraft, N., & Owen, N. (2015). Neighborhood environmental attributes and adults' sedentary behaviors: Review and research agenda. *Preventive Medicine*, *77*, 141–149.	Focus on sedentary behaviors as the main outcome without any parallel focus on PA related behaviors
Lee, H., Tamminen, K. A., Clark, A. M., Slater, L., Spence, J. C., & Holt, N. L. (2015). A meta-study of qualitative research examining determinants of children's independent active free play. *The International Journal of Behavioral Nutrition and Physical Activity*, *12*, 5.	No examination of the relationship between specific built environment features and PA outcomes

Masoumi, H. E. (2017). Associations of built environment and children's physical activity: a narrative review. *Reviews on Environmental Health*, *32*(4), 315–331.	Not a systematic search
Maurer Braun, L., Read, A., & Ricklin, A. (2016). The Benefits of Street-Scale Features for Walking and Biking. *Journal of Transport & Health*, *3*(2), S22. https://doi.org/10.1016/j.jth.2016.05.060	Process of the systematic search not detailed enough
Mitra, R. (2013). Independent Mobility and Mode Choice for School Transportation: A Review and Framework for Future Research. *Transport Reviews*, *33*(1), 21–43.	Not a systematic search
Ogilvie, D., Foster, C. E., Rothnie, H., Cavill, N., Hamilton, V., Fitzsimons, C. F., & Mutrie, N. (2007). Interventions to promote walking: Systematic review. *British Medical Journal*, *334*(7605), 1204–1207.	No examination of the relationship between specific built environment features and PA behaviors
Olsen, J. M. (2013). An integrative review of literature on the determinants of physical activity among rural women. *Public Health Nursing (Boston, Mass.)*, *30*(4), 288–311.	Very limited focus on the role of the built environment
Panter, J. R., & Jones, A. (2010). Attitudes and the Environment as Determinants of Active Travel in Adults: What Do and Don't We Know? *Journal of Physical Activity and Health*, *7*(4), 551–561.	This article could not be accessed
Park, K. (2017). Psychological park accessibility: a systematic literature review of perceptual components affecting park use. *Landscape Research*, *42*(5), 508–520.	Focus on park use as the main outcome. No specific PA behavior identified and examined
Rahman, T., Cushing, R. A., & Jackson, R. J. (2011). Contributions of Built Environment to Childhood Obesity. *Mount Sinai Journal of Medicine*, *78*(1), 49–57.	This article could not be accessed
Sallis, J. F., & Spoon, C. (2015). *Making the Case for Designing Active Cities*.	Not a systematic search
Sharma, M., Nahar, V.K. (2018). Promoting physical activity in upper elementary children using multi-theory model (MTM) of health behavior change. *J Prev Med Hyg* [Internet]. 59:E267–76. Available from: http://www.embase.com/search/results?subaction=viewrecord&from=export&id=L626004508	Not a systematic search
Siddiqi, Z., Tiro, J. A., & Shuval, K. (2011). Understanding impediments and enablers to physical activity among African American adults: A systematic review of qualitative studies. *Health Education Research*.	No focus on the built environment
Sterdt, E., Liersch, S., & Walter, U. (2014). Correlates of physical activity of children and adolescents: A systematic review of reviews. *Health Education Journal*, *73*(1), 72–89.	Review of reviews describing papers already selected
Stewart, G., Anokye, N. K., & Pokhrel, S. (2015). What interventions increase commuter cycling? A systematic review. *BMJ Open*, *5*(8), e007945.	Focus on interventions' designs. No focus on the relationship between the built environment and PA
Temple, M., & Robinson, J. C. (2014). A systematic review of interventions to promote physical activity in the preschool setting. *Journal for Specialists in Pediatric Nursing*, *19*(4), 274–284.	Very limited examination of built environment features
Trost, S. G., Ward, D. S., & Senso, M. (2010). Effects of child care policy and environment on physical activity. *Medicine and Science in Sports and Exercise*, *42*(3), 520–525.	Not a systematic search

Van Cauwenberghe, E., Maes, L., Spittaels, H., van Lenthe, F. J., Brug, J., Oppert, J.-M., & De Bourdeaudhuij, I. (2010). Effectiveness of school-based interventions in Europe to promote healthy nutrition in children and adolescents: systematic review of published and "grey" literature. *The British Journal of Nutrition*, *103*(6), 781–797.	No built environment component
Van Der Horst, K., Oenema, A., Ferreira, I., Wendel-Vos, W., Giskes, K., Van Lenthe, F., & Brug, J. (2007). A systematic review of environmental correlates of obesity-related dietary behaviors in youth. *Health Education Research*, *22*(2), 203–226.	No focus on the built environment. Availability and accessibility are not being explicitly linked to specific built environment features
Van Der Horst, K., Paw, M. J. C. A., Twisk, J. W. R., & Van Mechelen, W. (2007). A brief review on correlates of physical activity and sedentariness in youth. *Medicine and Science in Sports and Exercise*.	No examination of built environment components
Van Sluijs, E. M. F., McMinn, A. M., & Griffin, S. J. (2007). Effectiveness of interventions to promote physical activity in children and adolescents: systematic review of controlled trials. *BMJ.*, *335*(7622), 703.	No focus on the built environment
Verloigne, M., Van Lippevelde, W., Maes, L., Brug, J., & De Bourdeaudhuij, I. (2012). Family- and school-based correlates of energy balance-related behaviours in 10-12-year-old children: A systematic review within the ENERGY (EuropeaN Energy balance Research to prevent excessive weight Gain among Youth) project. *Public Health Nutrition*.	No focus on the built environment
Walker, R. E., Keane, C. R., & Burke, J. G. (2010). Disparities and access to healthy food in the United States: A review of food deserts literature. *Health and Place*, *16*(5), 876–884.	Focus on a SES perspective. Do not investigate the relationship between access to healthy food and its consumption
Wang, Y., Chau, C. K., Ng, W. Y., & Leung, T. M. (2016). A review on the effects of physical built environment attributes on enhancing walking and cycling activity levels within residential neighborhoods. *Cities*, *50*, 1–15. https://doi.org/10.1016/j.cities.2015.08.004	Process of the systematic search not detailed enough
Ward, D. S., Welker, E., Choate, A., Henderson, K. E., Lott, M., Tovar, A., … Sallis, J. F. (2017). Strength of obesity prevention interventions in early care and education settings: A systematic review. *Preventive Medicine*, *95 Suppl*, S37–S52.	No focus on the built environment
Yang, L., Sahlqvist, S., McMinn, A., Griffin, S. J., & Ogilvie, D. (2010). Interventions to promote cycling: Systematic review. *BMJ (Online)*.	No focus on the built environment
