# Supplementary material for: Built Environment and Health Behaviors: Deconstructing the Black Box of Interactions—A Review of Reviews
Source: Int J Environ Res Public Health. 2019 Apr 24;16(8):1454. doi: 10.3390/ijerph16081454 (PMC6517917; doi:10.3390/ijerph16081454)
Supplement: Supplementary file 1 [file ijerph-16-01454-s001.zip › ijerph-444662 Supplementary Materials - Final/Additional File 3 - Included records.pdf]

Supplementary Table S8 – Included records: description of Subset 1 and Subset 2.

	Records	Type	Date range	Included Studies	Aim & Objective	Population	Methodology	Key findings
Subset 1: coded articles included in the narrative synthesis (n=45)								
1	Adkins et al., 2017	PA	None	17	Examine the effect of the built environment on PA in different socioeconomic contexts	Socially disadvantaged population (low-income, racial minorities, poor)	Review	The observed effects of the built environment on PA seem to be weaker for disadvantaged than for advantaged population groups
2	Arango et al., 2013	PA	1990-6 Aug 2012	15	Examine the relationship between perceived environmental attributes and physical activity among Latin American adults	Adults & older adults in Latin America	Systematic Review	Safety was found to be related to leisure-time PA while street lightning was related to transport-related PA
3	Barnett et al., 2017	PA	Jan 2000 - 3 Sep 2016	100	Examine built environmental correlates of PA among older adults by type of PA and environmental attribute measurement	Older adults (≥65)	Systematic Review & Meta-Analysis	Walkability, safety, accessibility, availability, design and aesthetics were positively correlated to older adult's PA levels. Associations strength varied depending on PA types and environmental attributes measurement methodologies
4	Bauman et al., 2012	PA	1 Jan 1999 -	25	Examine the correlates and determinants of active leisure, recreation and active	Adults (≥18) and children (5-13) and	Review of Reviews	Multiple built environment attributes among which land-use mix and walkability were

			Apr 2012		transportation among children and adults in developed and developing countries	adolescents (12-18)		associated with PA. The strength of factors varied in function of age and the development situation of the country
5	Bloemen et al., 2015	PA	Jan 2000 - May 2013	18	Identify the key factors associated with PA in children and adolescent with physical disabilities	Children with a physical disability (aged 4-18)	Systematic Review	Adequate sport equipment and access to transport were found to be associated with PA in children with a physical disability
6	Carlin et al., 2017	PA	Jan 2004- Apr 2016	31	Identify the physical environmental determinants that influence PA across the life course	Children, Adolescents, Adults	Umbrella Systematic Review	Availability and safety of infrastructure and equipment were positively associated with physical activity in children and adolescents whereas negative street characteristics aspects were negatively associated with physical activity in adults
7	Casagrande et al., 2009	PA & Diet	-Jul 2007	10	Examine association of the built environment with PA and Diet among African Americans	African American Adults ≥18	Systematic Review	Light traffic, presence of sidewalks and safety from crime tend to be positively associated with PA. Presence of supermarkets and specialty stores positively associated with consumption of fruit and vegetables
8	Caspi et al., 2012	Diet	- March 2011	38	Evaluate the relationship between the food environment and diet & explore the different dimensions of “food access”	Adult, Children, Adolescent	Systematic Review	GIS-based measures were the most commonly used but were less consistently associated with diet than surveys and store audits

9	Cerin et al., 2017	PA	Jan 2000 – 6th Sep 2016	42	Examine and quantify the strength of evidence for environment-Active transport (AT) associations	Older adults (≥65)	Systematic Review & Meta-Analysis	Urban form features, good design and access were positively associated with AT walking whereas negative features such as neighborhood disorder were negatively associated with AT walking
10	Correa et al., 2015	Diet	2003-2013	90	Examine association between availability of food retailers and obesity in schoolchildren and adolescent	Schoolchildren, Adolescent	Narrative literature review	Distance to convenience stores was positively associated with healthier diets
11	D'Haese et al., 2015	PA	Jan 2000 - Aug 2014	65	Examine the relationship between six environmental variable groups and active travel to school	Children (6-12)	Systematic Review	Walkability, density and accessibility associated with walking for AT to school
12	De Craemer et al., 2012	PA & Diet	Jan 1990 – Sep 2010	43	Examine the correlates - notably the physical environment correlates - of physical activity, sedentary behavior and eating behaviors	Preschool children (4 to 6-year-old)	Systematic Review	Several physical environmental variables such as outdoor space and characteristics were correlated with higher levels of physical activity & accessibility to school (short distances, absence of barriers) was associated with higher levels of active transport to school
13	Ding et al., 2011	PA	-Jan 2010	103	Find neighborhood environment correlates of youth physical activity	Children (3-18)	Review	The strength of associations varied in function of the measurement methodologies employed

								Walkability, traffic speed and volume, access to recreational facilities, diversity and density were positively correlated to children PA<br>Land-use mix, and residential density were the strongest correlates for adolescents.
14	Eisenberg et al., 2017	PA	1990 - 2015	15	Examine the built environment as a potential moderator of the relationship between having a disability and lower levels of physical activity	Persons with disabilities (PWD)	Systematic Review	Findings suggests a positive moderating effect of safety, aesthetic and design on the PA levels of persons with disabilities
15	Engler-Stringer et al., 2014	Diet	1995 – July 2013	26	Evaluate the influence of the community and consumer nutrition environments on the diet of children	Children under age 18	Systematic Review	Objective and perceived measures of availability, accessibility and affordability showed that these factors play a role in defining the food environment and are associated with some characteristics of poor dietary intake
16	Escalante et al., 2014	PA	-31 May 2012	8	Examine built environment interventions aimed at increasing children's physical activity during school recess	Pre-schoolers school-children (2-12)	Systematic Review	Found that changes in playground markings together with an improvement of physical structures increase active recess among schoolchildren in the short to medium term

17	Feng et al., 2010	PA & Diet	(?) - 2008	63	Evaluate built environment-behavior (obesity related outcomes) associations across studies using different methodologies	General population	Systematic Review	Methodological heterogeneity could explain the absence of associations in some study
18	Fraser & Lock, 2011	PA	-Jun 2009	21	Examine the relationship between the built environment and cycling	General population	Systematic Review	Availability density proximity safety were identified as positively correlated to cycling. Destination proximity, safety, design barriers were identified as negative factors of cycling
19	Frerichs et al., 2015	Diet	-2015	102	Examine the relationship between school architecture and design on healthy eating	Children: kinder garden to 12th grade	Narrative systematic review	Serving water and vending machines access were found to have an impact on children's dietary choices
20	Frost et al., 2010	PA	-Jun 2008	20	Examine the influence of the built environment on adults' PA in rural settings	Adults (≥18) in rural settings	Review	Aesthetics, trails availability, destination accessibility and safety were found to be associated with PA in rural settings
21	Galvez et al., 2010	PA & Diet	Jan 2008 – Aug 2009	48	Examine the role of neighborhoods factors such as the built environment in childhood obesity and obesity related behaviors	Children (under 18)	Review	PA and diet outcomes varied across studies by individual and community level characteristics. Community level factors such as features promoting active commuting and walkability were found to be linked to individual's behaviors but are not yet specifically detailed in the Ecological System Theory Model

22	Giskes et al., 2011	Diet	Jan 2005 – Oct 2008	28	Examine the association between physical environmental factors and obesogenic dietary behaviors	Adults > 18	Systematic Review	Accessibility factors play a significant role in the relationship between the physical environment and diet related behaviors
23	Glanz et al., 2012	Diet	1995 - 2010	125	Examine the relationship between grocery store marketing strategies modifying the built environment and diet-related behaviors such as healthful eating	Consumers	Review	Increasing availability, affordability, prominence and promotion of healthful foods and/or restricting the marketing of unhealthy foods was found to increase healthful eating
24	Grasser et al., 2013	PA	-Aug 2010	34	Examine 4 GIS-based measures of walkability (density, land-use mix, connectivity and walkability indexes) in urban and suburban settings and their association with active transportation	Healthy white adults (> 19) living in urban and suburban neighborhoods	Systematic Review	Population density, intersection density and walkability indexes were positively associated with active travel
25	Kaczynski & Henderson, 2007	PA	1998 - 2005	50	Examine the relationship between parks and recreation settings - as features of the built environment - and physical activity	Parks and recreation facilities users	Review	Parks and recreation accessibility was generally found to be associated with increased physical activity
26	Kahn-Marshall & Gallant, 2012	PA & Diet	1995 - 2010	27	Examine - among other things - the effectiveness of worksite health promotion	Work-site employee	Systematic Review	Increased desirability, affordability and availability of healthy food options showed

					programs such as physical environment changes on employees' physical activity and diet related behaviors in the workplace			positive impacts on dietary behaviors & stair prompts, motivational signs, skip-stop elevators and stairwells modifications were linked to increased physical activity
27	Kaushal & Rhodes, 2014	PA	-May 2014	49	Examine the relationship between the home physical environment and adults/children's PA and sedentary behaviors	Children, Adults	Systematic Review	Large exercise equipment had a greater impact on PA than small ones. Quantity of these equipment was correlated with PA in girls. Availability of TVs was more strongly correlated with sedentary behaviors in girls
28	Kraak et al., 2017	Diet	Jan 2000 - Dec 2016	5	Examine the effect of nudges and choice architecture strategies aimed at promoting healthy food environments and healthy diets in restaurant settings	General population	Systematic review	Identified 5 frameworks of choice architecture deconstructing the mechanisms of nudges on diet-related behaviors
29	Krolner et al., 2011	Diet	-Dec 2010	31	Examine qualitative evidence of determinants of children's fruit and vegetable (FV) intake	Children 6 to 18-year-old	Systematic Review	Physical environment factors such as eating settings, visibility of and access to unhealthy food have been identified as influencing children's FV consumption
30	Larson et al., 2009	Diet	1985 – Apr 2008	54	Examine neighborhood differences in access to food	Neighbor-hood residents	Review	Residents with better access to supermarkets and limited access to convenience stores were found to have healthier diets.

31	Lawman & Wilson, 2012	PA & Diet	1995 - 2010	38	Examine among other environmental factors the role of the neighborhood environment on physical activity, diet and sedentariness in youth at high risk for chronic disease	At risk youths (aged 10 to 18)	Review	Home and neighborhood accessibility and safety were generally found to be significantly related to health behaviors. Availability of neighborhood resources for PA might increase the use of these resources although it may not be related to general PA. Moderators of this relationship are to be considered
32	Limstrand, 2008	PA	-Nov 2006	43	Identify the role of the relevant factors playing a role in the relationship between sports facilities and youths' PA	Children and adolescents (2-19)	Review	Access and user characteristics were found to be consistently correlated to youths’ PA
33	Lovasi et al., 2009	PA	1 Jan 1995 - 27 Jan 2009	45	Examine the relationship between the built environment and obesity-related heath disparities	Disadvantages individuals or areas with low SES, or minorities (Black or Hispanic)	Review	Supermarkets (instead of smaller grocery or convenience stores), places to exercise and safety were found to be potentially positively influential for the diet of the identified disadvantaged groups
34	Lu et al., 2014	PA	Feb 2013	39	Examine the perceived barriers to children’s active commuting to school	Children and adolescents (4-19) and or related adults (parents and teachers)	Systematic Review	Perceived traffic safety and perceived distance were among the most cited factors associated with children not actively commuting to school

35	Mayne et al., 2015	PA & Diet	2005-2013	37	Examine different methodologies and study designs that evaluate the impact of policy and built environment changes on obesity-related outcomes	General population	Systematic review	Improvements made to active transportation infrastructures have a positive impact on PA, notably cycling. Food availability was associated to consumption
36	McCormack & Shiell, 2011	PA	-	33	Examine the relationship between the built environment and physical activity by taking into accounts the mechanism of self-selection	Adults (≥18)	Systematic Review	The built environment was more likely to be associated to AT walking rather than other PA types and only a few studies found that residential self-selection attenuated the association
37	McGrath et al., 2015	PA	Jan 2000-Mar 2013	23	examine the relationship between specific built environment attributes and children's and adolescents' PA levels (moderate-vigorous PA, including walking)	Children, Adolescents (5-17)	Systematic Review & Meta-Analysis	Play facilities, parks, playgrounds and street designs promoting walking had positive effects on adolescents PA but negative effects on children's PA
38	Mhurchu et al., 2013	Diet	-31 Dec 2012	39	Examine the associations between consumer and community food environments and dietary outcomes	General population	Review	The influence of the availability of healthy foods on diets is moderated by the concurrent availability of unhealthy food
39	Moran et al., 2014	PA	1996-2012	31	Examine the relationship between the physical environment and older adults' PA	Adults (≥65)	Review	Access to facilities, green open spaces and rest areas was found particularly relevant to explain older adults' PA behaviors

40	Moschonis et al., 2015	PA & Diet	Jan 1993-Apr 2013	81	Develop a conceptual framework presenting the environmental and personal determinants of cardio-metabolic risk behaviors in childhood	Children (6 to 12)	Review	Availability, accessibility, safety and aesthetics of suitable infrastructures were found to influence cardio-metabolic related behaviors among children and adolescents
41	Pucher et al., 2010	PA	1990-(?)	139	General population	General population	Review	The more people cycling, the highest the perception of safety ("safety in numbers"). Biker characteristics also play a role in the studied relationship
42	Saelens & Handy, 2008	PA	2005-May 2006	42	Examine the built environment correlates of walking	General population	Review	Positive relations found between walking for transportation and density, accessibility and diversity
43	Shields et al., 2012	PA	-Sep 2012	14	Examine the perceived barriers and facilitators to PA in children with disability	Children (<18) with disability	Systematic Review	Accessibility of facilities were notably found to impact PA levels in children with disability
44	Sugiyama et al., 2012	PA	-2011	46	Examine the association of destination and route attribute with utilitarian and recreational walking	Adults	Review	Utilitarian walking was associated with availability and accessibility of retail and service destinations as well as with route design (functional aspects). Route aesthetics and the availability, proximity and quality of destinations was associated with recreational walking

45	Yen et al., 2014	PA	1991-2011	123	Examine how place influences older adult's decision about mobility, including PA levels	Adults (≥50)	Review	Safety was at the hinge of the relationship between built environmental components such as connectivity, aesthetics and the decision of being physically active
Subset 2: non-coded articles included in the narrative synthesis (n=62)								
46	Al-Khudairy et al. 2019	Diet	-May 2017	18	Examine relevant interventions using choice architecture to improve diet among healthcare staff	Healthcare staff of high income countries	Systematic Review	Choice architecture interventions focusing on proximity, availability and sizing were found to be effective on healthcare staff
47	An, Shen, Yang, & Yang, 2019	PA	31 May 2018	20	Examine the influence of built environment features and characteristics on PA and obesity among youths in China	Chinese children and/or adolescents aged ≤17	Narrative Systematic Review	Increased PA levels and reduced sedentary behaviors were found to be associated with the availability and accessibility of built environment features such as parks, greenspaces, sidewalks and recreational facilities
48	Annear et al., 2014	PA	-	83	Examine the influence of environmental factors on older adults' health and active ageing	Older adults (≥50)	Systematic Review	Street lightning, traffic, accessibility to facilities, aesthetics, safety, density and infrastructure design were found to influence older adults' active ageing
49	Audrey & Batista-Ferrer, 2015	PA	- 29 Oct 2014	33	Examine urban environment interventions aimed at increasing healthy behaviors such as PA among youths	Children	Systematic Review	Road safety and active travel measures were found to be associated with physical activity among youths
50	Bancroft et al., 2015	PA	Jan 1990 -	20	Examine the relationship between parks proximity and	General USA population	Systematic Review	Mixed findings due to measurement heterogeneity. Five

			Jun 2013		density and physical activity among the USA population			studies found that parks access was positively associated with PA
51	Bellicha et al., 2015	PA	-mid 2013	60	Examine the effectiveness of stair-use interventions to promote PA on worksites	Worksite population	Systematic Review	The combination of motivational and directional signs led to a strong increase in stair climbing
52	Broekhuizen, Scholten, & de Vries, 2014	PA	Jan 2000 - Sept 2012	30	Examine the effect of preschool playgrounds on children's health, notably in terms of physical activity	Children (2-18)	Systematic Review	Availability of playground equipment was found to be associated with PA among children
53	Bull F, Hutton C, Cavill N, 2007	PA	Jan 1990 - Jul 2006	10	Examine the effect of built environment interventions on physical activity	Employees, children and others	Systematic Review	Availability and aesthetics had a positive impact on PA in the workplace. Aesthetics had a positive impact on stair use and playgrounds use during school recess. Aesthetics and availability of equipment in the classroom also had a positive impact on PA
54	Dadpour, Pakzad, & Khankeh, 2016	PA	1990 - 9 Nov 2015	10	Examine the influence of the environment on adult's walking	Adults (18-65)	Meta-Synthesis	Safety, aesthetics, convenience and efficiency were found to influence adult's walking
55	Ding et al., 2018	PA	-Mar 2017	23	Examine the influence of neighborhood environmental changes on PA, walking and travel behavior through the effects residential relocation	General population	Systematic Review	Increased walkability of the neighborhood environment was found to be associated with increased levels of walking among relocated populations
56	Dobbins, Maureen, Tirilis, 2011	PA	1985 - May 2011	27	Examine the effectiveness of population-based interventions on the built environment in relation to health outcomes, notably PA	General population, children, adults and ethnic and low-income populations	Review of reviews	Street design, safety and aesthetics were found to enhance PA

57	Durand, Andalib, Dunton, Wolch, & Pentz, 2011	PA	2000 - mid Mar 2009	44	Review the built environment factors associated with PA and obesity risk and their implications for smart growth urban planning	General population	Systematic Review	Built environment attributes also identified as smart growth factors, namely diverse housing types, mixed land use, housing density, compact development patterns and levels of open space were associated with increased PA levels mostly related to walking
58	Ewing & Cervero, 2010	PA	-2009	61	Examine the relationship between built environment factors and active travel in order to draw generalizable conclusions for practice	General population	Meta-Analysis	Land use diversity, intersection density, destination availability and accessibility were found to be related to walking.<br>Transit proximity, street network design and land use diversity were related to bus and train use
59	Farkas, Wagner, Nettel-Aguirre, Friedenreich, & McCormack, 2019	PA	-31 Dec 2016	25	Examine the associations between objectively measured aspects and features of the built environment and walking among Canadian adults	Canadian adults (≥18)	Systematized Review	Specific behaviors such as walking for transportation were found to be associated with overall walkability and land use whereas proximity to destination was associated with walking for any purpose
60	Hajna et al., 2015	PA	-20 May 2014	6	Examine the association between neighborhood walkability and PA (walking measured by biosensor daily steps) in adults	Adults (≥18)	Systematic Review & Meta-Analysis	Adults living in more walkable neighborhoods had higher levels of PA
61	Hollands et al., 2013	PA & Diet	1948-Jun 2011	440	Examine the effects of choice architecture interventions in micro-environment on health-related behaviors,	General population	Scoping Review	Food accessibility, proximity, portion sizing and aesthetics improvements were consistently found to be associated with healthy diet outcomes. Prompting

					notably diet and physical activity			and priming where also found to have a positive impact both on PA and diet
62	Hunter et al., 2015	PA	-July 2014	12	Examine the effectiveness of interventions promoting PA in urban green space	General population	Systematic Review	An association was found between urban green space and PA levels in the population. Interventions combining built environment modification and activity programs were found to have a positive impact on PA levels
63	Lachowycz & Jones, 2011	PA	2000-2010	60	Examine the association between objectively measured access to greenspace and obesity-related outcome, including PA	General population	Systematic Review	The association between greenspace and PA was positive but varied in function of personal factors such as age or socioeconomic status (SES)
64	A. C. K. Lee & Maheswaran, 2011	PA	1990 - Jun 2010	35	Examine the positive health impacts of urban green spaces and public open spaces	High income country population	Review	Accessibility and quality of parks were identified as factors that affect their use for PA along with other personal factors such as age, gender or ethnicity that tend to shape the perception of safety
65	L.-L. Lee, Kuo, & Chan, 2016	PA	-	5	Examine the associations between attributes of the built environment and physical activity in adolescents dwelling in East Asia	East Asian Adolescents (10-19)	Systematic Review	Barriers to public facilities accessibility was negatively associated with PA. Gender differences were also notices between boys and girls
66	Maitland, Stratton, Foster, Braham, & Rosenberg, 2013	PA	2005-2011	49	Examine the influence of the home physical environment on children's PA	Children (8-14)	Systematic Review	The availability of media equipment was associated with sedentary behaviors. PA equipment in the home was not

								associated with PA although it was inversely associated with sedentary behaviors
67	Malambo, Kengne, De Villiers, Lambert, & Puoane, 2016	PA	2005-Apr 2015	18	Examine the influence of built environmental attributes on cardiovascular disease outcomes	Adults (≥18)	Systematic Review	Several built environment attributes such as residential density, safety from traffic, recreational facilities, street connectivity and high walkable environment were found to be associated with PA
68	McCormack, Rock, Toohey, & Hignell, 2010	PA	1995-2008	21	Examine qualitative evidence of the association of urban parks characteristics with use and PA	General population	Review	Safety, aesthetics, amenities, maintenance and proximity were found to be associated with park use. Perceptions of the physical environment were strongly related with perceptions of the social environment
69	McCrorie, Fenton, & Ellaway, 2014	PA	-12th June 2013	14	Examine the relationship between the environment and objectively measured PA in children	Children (5-18)	Review	Roads, streets, school grounds and the home location were found to be important locations for PA. Children expose to green spaces tend to be more physically active
70	Nocon, Muller-Riemenschneider, Nitzschke, & Willich, 2010	PA	Jan 2001 - May 2008	25	Examine the effectiveness of point-of-choice prompt in increasing PA	General population	Systematic Review	Point-of-choice prompts were effective in increasing stair climbing, particularly in escalator settings
71	Oliveira, Moreira, Abreu, Mota, & Santos, 2014	PA	Jan 2007-Jan 2012	28	Examine the amount and type of environmental features or attributes influencing PA in children	Children (3-12)	Systematic Review	Sidewalks and bike lanes were positively related to PA

72	Ikeda, Hinckson, Witten, & Smith, 2018	PA	Jan 2000- Jul 2017	37	Examine the associations between perceived physical environment aspects/features, sociodemographic characteristics and school travel modes in children	Children aged 5 to 13	Systematic Review	Safety and walkability were found to positively influence active school travelling
73	Karmeniemi, Lankila, Ikaheimo, Koivumaa-Honkanen, & Korpelainen, 2018	PA	-Dec 2015	51	Examine the influence of changes in the built environment on changes in behaviors pertaining to PA	General population	Systematic Review	Both perceived and objective changes in the built environment were positively associated with changes in PA
74	Levy-Storms, Chen, & Loukaitou-Sideris, 2017	PA	Jan 1970- Dec 2015	48	Examine older adults' preferences and needs related to their PA in and near open spaces such as parks	Older adults (≥65)	Systematic Review	Older adults have some specific needs and preferences pertaining to open spaces, that influence their behavior related to PA
75	Luiu, Tight, & Burrow, 2018	PA	1991-	54	Examine the transport barriers influencing active travelling	Older adults	Scoping review	Built environment barriers to active travel for older adults mitigate the benefits provided by facilitators such as the availability of public transport or flexible transport services
76	MacMillan et al., 2018	PA & Diet	-May 2017	19	Examine the effects of neighborhood built environment changes on PA and diet in residents	General population	Systematic Review	The development of cycle/walking trails, rails stops/lines, supermarkets, farmers' markets, park and green spaces was found to be positively associated with increased PA and healthier diet outcomes

77	Marzi, Demetriou, & Reimers, 2018	PA	Jan 1990-Nov 2017	27	Give an overview of socio-ecological correlates of independent mobility in children with a specific focus on sex and gender differences	Healthy children aged 3-12 (or with an average age in this range)	Systematic Review	Both the physical and the social environment were found to influence children's independent mobility. The social environment was found to be more influential, however, many aspects examined, such as the perception of traffic or fear of crime and stranger, were pertaining to both the physical environment and the social environment
78	Panter, Jones, & van Sluijs, 2008	PA	-	24	Examine the perceived and objectively measured environmental determinants of active travel in youth	Children (5-18)	Systematic Review	Road safety, distance and urban form were found to influence children's active travel (walking and cycling)
79	Parrish, Okely, Stanley, & Ridgers, 2013	PA	Jan 2000-Apr 2011	9	Examine the effects of recess-based interventions youths' PA	Children (5-18)	Systematic Review	Playground markings and games equipment were found to increase recess and lunchtime PA
80	Pont, Ziviani, Wadley, Bennett, & Abbott, 2009	PA	1985 - May 2008	38	Examine the environmental correlates of active transportation in youths	Children (5-18)	Systematic Review	Distance was negatively associated with youths' PA
81	Rahmanian, Gasevic, Vukmirovich, & Lear, 2014	Diet	Jan 2000-Sept 2013	24	Examine the association between the built environment and diet	Adults (≥18)	Systematic Review	Accessibility was generally associated with greater consumption of fruits and vegetables
82	Renalds, Smith, & Hale, 2010	PA	Jan 2003-Jan 2009	23	Examine the relationship between the built environment and health	General population	Systematic Review	An association was found between walkable neighborhoods and increased PA
83	Rhodes, Saelens, & Sauvage-Mar, 2018	PA	-Oct 2017	22	Examine the interaction between the built	General population	Systematic Review	Several aspects of the built environment were found to play a role in the interactions with PA:

					environment and social cognition to predict PA			accessibility and convenience were found to be positively related to PA intention whereas perceived aspects such as aesthetics were found to negatively influence leisure-time PA
84	Ridgers, Salmon, Parrish, Stanley, & Okely, 2012	PA	Jan 1990- Apr 2011	53	Examine the correlates of school recess PA among youths	Children (5-18)	Systematic Review	Availability of facilities and equipment were associated with recess PA
85	Rosso, Auchincloss, & Michael, 2011	PA	1 Jan 1990- 7 Dec 2010	17	Examine the relationship between the objectively measured built environment and older adults' mobility	Adults (18-60)	Review	Street connectivity, short distances, traffic, safety, destinations (pars and green spaces) were associated with mobility among older adults
86	Rothman, Macpherson, Ross, & Buliung, 2018	PA	Jan 1990- 30 Jul 2016	63	Examine the correlates of active travel to school in North America	Children 4-12	Systematic Review	Distance to school was associated with active travel to school. Individual, parental and societal correlates were also found to have moderate positive associations with AST
87	Salvo, Lashewicz, Doyle-Baker, & McCormack, 2018	PA	-Apr 2016	36	Analyzes the influences of the built environment on PA among adults, based on qualitative evidence	Adults	Systematized Review	Sociodemographic characteristics play a role in the relationship between the built environment and PA
88	Sawyer, Ucci, Jones, Smith, & Fisher, 2017	PA	1980 - Feb 2016	46	Examine the combined influence of neighborhood physical and social environments on PA and explore the interactions at play in this relationship	Adults (≥15)	Systematic Review	Physical and social environment features were found to play a simultaneous role on PA levels

89	Schüle & Bolte, 2015	PA	-5th Nov 2013	33	Examine both socioeconomic and built environment factors' influence on PA and identify their respective role and independent interactive effects in the association with PA	General population	Systematic Review	Interactions were found between the social and the built environment as well as with individual characteristics such as sex or ethnicity
90	Schulz, Romppel, & Grande, 2018	PA & Diet	1990- Sep 2016	25	Examine the relationship between the built environment and leading risk factors and health behaviors in Germany	General German population	Systematic Review	Access was found to be associated with sport-related PA
91	Sharmin & Kamruzzaman, 2017	PA	Jan 1980 - May 2016	12	Examine the built environment impact on children independent mobility	Adults (≥18)	Meta-Analytic Review	Dead-end streets were found to have the strongest positive association with children independent mobility among all the associations examined by the authors whereas land-use mix was the strongest negative association
92	Sirard & Slater, 2008	PA	1975- Mar 2007	40	Examine, among other things, the correlates of active commuting to school	Children	Review	Parents' perceptions of their physical and social environment influence children's active commuting levels
93	Smith et al., 2017	PA	-Jun 2015	28	Examine the effectiveness of environmental interventions to increase PA and active transport	General population	Systematic Review	The evidence suggests that infrastructure improvements may mostly benefit to socioeconomically advantaged groups. There was a positive effect of walkability, availability of parks and playgrounds and improvements in active transport infrastructures on PA levels

94	Soler et al., 2010	PA	-20 Apr 2005	16	Examine the effectiveness of point-of-decision prompts and enhancements to stairwells to increase stair use	General population	Systematic Review	The interventions were followed by significant increases in stair use
95	Stanley, Ridley, & Dollman, 2012	PA	1990-Jan 2011	22	Examine the correlates of children's PA during school recess and after-school periods	Children (8-14)	Review	Access to equipment, playground markings and play space were associated with PA
96	Stappers, Van Kann, Ettema, De Vries, & Kremers, 2018	PA	-Feb 2018	19	Examine the relationship between built environment infrastructural changes and PA, including active transportation and sedentary behavior	Adults (≥18)	Systematic Review	Bicycling was positively associated with proximity to the infrastructures
97	Starnes, Troped, Klenosky, & Doehring, 2011	PA	1980-2008	52	Examine the relationship between trails' features and PA	General population	Review	Land-use mix and distance to trail were related to trail use
98	Stewart, 2011	PA	2000-2008	42	Examine factors associated with active transportation to school	Children (≤18)	Review	Built environment factors such as distance and traffic were found to frequently influence active transportation to school
99	Van Cauwenberg et al., 2011	PA	Jan 2000-Mar 2010	31	Examine the relationship between the physical environment and PA in older adults	Older adults (≥65)	Systematic Review	Only a few environmental characteristics were related to total PA, which suggests the need to analyze specific built environment features in relation to specific PA related outcomes
100	Van Cauwenberg et al., 2018	PA	1 Jan 2000-15 Dec 2017	72	Examine the relationship between the built environment and leisure-time PA in older adults	Older adults (≥65)	Systematic Review & Meta-Analysis	Positive associations were found between leisure-time PA and walkability, land use mix access and aesthetics

101	Van Hecke et al., 2018	PA	Jan 2000-Apr 2017	31	Examine the relationship between adolescent public open spaces visitation/PA and the built environment characteristics of these places	Adolescents (12-16)	Systematic Review	A positive association was found between public open space visitation/PA and the availability of specific built environment features such as trails, playgrounds or sports fields
102	Van Holle et al., 2012	PA	Jan 2000-Aug 2011	70	Examine Europe-specific evidence on the associations between physical environment features and different PA domains in adults	Adults (18-65)	Systematic Review	Walkability, access to destinations and environmental quality were found to be positively related to PA in adults. The strength of the relationship between the built environment and PA varied in function of the PA domain
103	Wendel-Vos, Droomers, Kremers, Brug, & van Lenthe, 2007	PA	1 Jan 1980-31 Dec 2004	47	Examine the potential determinants of different PA levels in adults	Adults (≥18)	Systematic Review	Availability of equipment was found to be associated with PA/sports and connectivity of trails with active commuting
104	Williams et al., 2014	Diet	-Oct 2013	30	Examine associations between the food environment (food outlets proximity to schools) and outcomes such as children's food purchases and consumption	Schoolchildren (5-18)	Systematic Review	Little evidence was found of an effect of fast food proximity to schools with schoolchildren food purchase and consumption
105	Wong, Faulkner, & Buliung, 2011	PA	-May 2010	14	Examine the relationship between objectively measured built environment features and active school travel in children and adolescents	Children, Adolescents (5-18)	Systematic Review	Distance was consistently found to be negatively associated with active school travel
106	Zapata-Diomedi & Veerman, 2016	PA	2009-15	23	Examine Australian evidence for the association between	Adults (≥18)	Systematic Review	Walkable neighborhood, destination diversity and land-

			Mar 2015		the built environment and physical activity in adults			use mixed were found to be related to increased PA
107	Zhang, Wulff, Duan, & Wagner, 2019	PA	2008-2016	25	Examine the influence of built environment characteristics of park and their neighborhood on park-based PA	General population	Systematic Review	The availability of paths/trails, lighting and the absence of broken glasses and litter were found to be positively associated with park-based PA

References

Adkins, A., Makarewicz, C., Scanze, M., Ingram, M., & Luhr, G. (2017). Contextualizing Walkability Do Relationships Between Built Environments and Walking Vary by Socioeconomic Context? *Journal of the American Planning Association*, *83*(3), 296–314. https://doi.org/10.1080/01944363.2017.1322527

Al-Khudairy, L., Uthman, O. A., Walmsley, R., Johnson, S., & Oyebode, O. (2019). Choice architecture interventions to improve diet and/or dietary behaviour by healthcare staff in high-income countries: A systematic review. *BMJ Open*, *9*(1). https://doi.org/10.1136/bmjopen-2018-023687 LK

An, R., Shen, J., Yang, Q., & Yang, Y. (2019). Impact of built environment on physical activity and obesity among children and adolescents in China: A narrative systematic review. *Journal of Sport and Health Science*. https://doi.org/10.1016/j.jshs.2018.11.003 LK

Annear, M., Keelings, S., Wilkinson, T. I. M., Cushman, G., Gidlow, B. O. B., & Hopkins H. (2014). Environmental influences on healthy and active ageing: a systematic review. *Ageing and Society*, *34*(4), 590–622. https://doi.org/10.1017/S0144686X1200116X

Arango, C. M., Paez, D. C., Reis, R. S., Brownson, R. C., & Parra, D. C. (2013). Association between the perceived environment and physical activity among adults in Latin America: a systematic review. *The International Journal of Behavioral Nutrition and*

Physical Activity, *10*, 122. https://doi.org/10.1186/1479-5868-10-122
Audrey, S., & Batista-Ferrer, H. (2015). Healthy urban environments for children and young people: A systematic review of intervention studies. *Health & Place*, *36*, 97–117. https://doi.org/10.1016/j.healthplace.2015.09.004
Bancroft, C., Joshi, S., Rundle, A., Hutson, M., Chong, C., Weiss, C. C., … Lovasi, G. (2015). Association of proximity and density of parks and objectively measured physical activity in the United States: A systematic review. *Social Science & Medicine (1982)*, *138*, 22–30. https://doi.org/10.1016/j.socscimed.2015.05.034
Barnett, D. W., Barnett, A., Nathan, A., Van Cauwenberg, J., Cerin, E., & Grp, C. O. A. W. (2017). Built environmental correlates of older adults' total physical activity and walking: a systematic review and meta-analysis. *International Journal of Behavioral Nutrition and Physical Activity*, *14*. https://doi.org/10.1186/s12966-017-0558-z
Bauman, A. E., Reis, R. S., Sallis, J. F., Wells, J. C., Loos, R. J. F., & Martin, B. W. (2012). Correlates of physical activity: Why are some people physically active and others not? *The Lancet*, *380*(9838), 258–271. https://doi.org/10.1016/S0140-6736(12)60735-1
Bellicha, A., Kieusseian, A., Fontvieille, A.-M., Tataranni, A., Charreire, H., & Oppert, J.-M. (2015). Stair-use interventions in worksites and public settings - a systematic review of effectiveness and external validity. *Preventive Medicine*, *70*, 3–13. https://doi.org/10.1016/j.ypmed.2014.11.001
Bloemen, M. A. T., Backx, F. J. G., Takken, T., Wittink, H., Benner, J., Mollema, J., & de Groot, J. F. (2015). Factors associated with physical activity in children and adolescents with a physical disability: a systematic review. *Developmental Medicine and Child Neurology*, *57*(2), 137–148. https://doi.org/10.1111/dmcn.12624
Broekhuizen, K., Scholten, A.-M., & de Vries, S. I. (2014). The value of (pre)school playgrounds for children's physical activity level: a systematic review. *The International Journal of Behavioral Nutrition and Physical Activity*, *11*, 59. https://doi.org/10.1186/1479-5868-11-59
Bull F, Hutton C, Cavill N, F. C. (2007). Physical activity and the environment review five: building design review. *NICE*, *8*(London), 1–71. Retrieved from https://www.nice.org.uk/guidance/ng90/evidence/building-design-evidence-review-pdf-4787281407
Carlin, A.; Perchoux, C.; Puggina, A.; Aleksovska, K.; Buck, C. . B., & C.; Cardon, G.; Chantal, S.; Ciarapica, D.; Condello, G.;

Coppinger, T.; Cortis, C.; D'Haese, S.; De Craemer, M.; Di Blasio, A.; Hansen, S.; Iacoviello, L.; Issartel, J.; Izzicupo, P.; Jaeschke, L.; Kanning, M.; Kennedy, A.; Lakerveld, J.; Ling, F.C.M.; L, S. (2017). A life course examination of the physical environmental determinants of physical activity behaviour: A "Determinants of Diet and Physical Activity" (DEDIPAC) umbrella systematic literature review. *PLoS ONE*, *12*(8). https://doi.org/10.1371/journal.pone.0182083

Casagrande, S. S., Whitt-Glover, M. C., Lancaster, K. J., Odoms-Young, A. M., & Gary, T. L. (2009). Built environment and health behaviors among African Americans: a systematic review. *American Journal of Preventive Medicine*, *36*(2), 174–181. https://doi.org/10.1016/j.amepre.2008.09.037

Caspi, C. E., Sorensen, G., Subramanian, S. V, & Kawachi, I. (2012). The local food environment and diet: a systematic review. *Health & Place*, *18*(5), 1172–87. https://doi.org/10.1016/j.healthplace.2012.05.006

Cerin, E., Nathan, A., van Cauwenberg, J., Barnett, D. W., & Barnett, A. (2017). The neighbourhood physical environment and active travel in older adults: a systematic review and meta-analysis. *The International Journal of Behavioral Nutrition and Physical Activity*, *14*(1), 15. https://doi.org/10.1186/s12966-017-0471-5

Correa, E. N., Soares Schmitz, B. de A., & de Vasconcelos, F. de A. (2015). Aspects of the built environment associated with obesity in children and adolescents: A narrative review. *Revista de Nutricao-Brazilian Journal of Nutrition*, *28*(3), 327–340. https://doi.org/10.1590/1415-52732015000300009

D'Haese, S., Vanwolleghem, G., Hinckson, E., De Bourdeaudhuij, I., Deforche, B., Van Dyck, D., & Cardon, G. (2015). Cross-continental comparison of the association between the physical environment and active transportation in children: a systematic review. *The International Journal of Behavioral Nutrition and Physical Activity*, *12*, 145. https://doi.org/10.1186/s12966-015-0308-z

Dadpour, S., Pakzad, J., & Khankeh, H. (2016). Understanding the Influence of Environment on Adults' Walking Experiences: A Meta-Synthesis Study. *International Journal of Environmental Research and Public Health*, *13*(7). https://doi.org/10.3390/ijerph13070731

De Craemer, M., De Decker, E., De Bourdeaudhuij, I., Vereecken, C., Deforche, B., Manios, Y., & Cardon, G. (2012). Correlates of energy balance-related behaviours in preschool children: A systematic review. *Obesity Reviews*. https://doi.org/10.1111/j.1467-

789X.2011.00941.x

Ding, D., Sallis, J. F., Kerr, J., Lee, S., & Rosenberg, D. E. (2011). Neighborhood Environment and Physical Activity Among Youth A Review. *American Journal of Preventive Medicine*, *41*(4), 442–455. https://doi.org/10.1016/j.amepre.2011.06.036

Ding, D., Nguyen, B., Learnihan, V., Bauman, A. E., Davey, R., Jalaludin, B., & Gebel, K. (2018). Moving to an active lifestyle? A systematic review of the effects of residential relocation on walking, physical activity and travel behaviour. *British Journal of Sports Medicine*, *52*(12), 789+. https://doi.org/10.1136/bjsports-2017-098833

Dobbins, Maureen; Tirilis, D. (2011). Commissioned Reports Built Environment : A Synthesis of Review of Evidence, (3).

Durand, C. P., Andalib, M., Dunton, G. F., Wolch, J., & Pentz, M. A. (2011). A systematic review of built environment factors related to physical activity and obesity risk: implications for smart growth urban planning. *Obesity Reviews : An Official Journal of the International Association for the Study of Obesity*, *12*(5), e173-82. https://doi.org/10.1111/j.1467-789X.2010.00826.x

Eisenberg, Y., Vanderbom, K. A., & Vasudevan, V. (2017). Does the built environment moderate the relationship between having a disability and lower levels of physical activity? A systematic review. *Preventive Medicine*, *95S*, S75–S84. https://doi.org/10.1016/j.ypmed.2016.07.019

Engler-Stringer, R., Le, H., Gerrard, A., & Muhajarine, N. (2014). The community and consumer food environment and children's diet: A systematic review. *BMC Public Health*. https://doi.org/10.1186/1471-2458-14-522

Escalante, Y., Garcia-Hermoso, A., Backx, K., & Saavedra, J. M. (2014). Playground designs to increase physical activity levels during school recess: a systematic review. *Health Education & Behavior : The Official Publication of the Society for Public Health Education*, *41*(2), 138–144. https://doi.org/10.1177/1090198113490725

Ewing, R., & Cervero, R. (2010). Travel and the built environment. *Journal of the American Planning Association*, *76*(3), 265–294. https://doi.org/10.1080/01944361003766766

Farkas, B., Wagner, D. J., Nettel-Aguirre, A., Friedenreich, C., & McCormack, G. R. (2019). A systematized literature review on the associations between neighbourhood built characteristics and walking among Canadian adults. *Health Promotion and Chronic Disease Prevention in Canada Research Policy and Practice*, *39*(1), 1–14. https://doi.org/10.24095/hpcdp.39.1.01

Feng, J., Glass, T. A., Curriero, F. C., Stewart, W. F., & Schwartz, B. S. (2010). The built environment and obesity: A systematic review of the epidemiologic evidence. *Health and Place*, *16*(2), 175–190. https://doi.org/10.1016/j.healthplace.2009.09.008

Fraser, S. D. S., & Lock, K. (2011). Cycling for transport and public health: a systematic review of the effect of the environment on cycling. *European Journal of Public Health*, *21*(6), 738–743. https://doi.org/10.1093/eurpub/ckq145

Frerichs, L., Brittin, J., Sorensen, D., Trowbridge, M. J., Yaroch, A. L., Siahpush, M., … Huang, T. T.-K. (2015). Influence of school architecture and design on healthy eating: a review of the evidence. *American Journal of Public Health*, *105*(4), e46-57. https://doi.org/10.2105/AJPH.2014.302453

Frost, S. S., Goins, R. T., Hunter, R. H., Hooker, S. P., Bryant, L. L., Kruger, J., & Pluto, D. (2010). Effects of the built environment on physical activity of adults living in rural settings. *American Journal of Health Promotion : AJHP*, *24*(4), 267–283. https://doi.org/10.4278/ajhp.08040532

Galvez, M. P., Pearl, M., & Yen, I. H. (2010). Childhood obesity and the built environment. *Current Opinion in Pediatrics*, *22*(2), 202–207. https://doi.org/10.1097/MOP.0b013e328336eb6f

Giskes, K., van Lenthe, F., Avendano-Pabon, M., & Brug, J. (2011). A systematic review of environmental factors and obesogenic dietary intakes among adults: are we getting closer to understanding obesogenic environments? *Obesity Reviews : An Official Journal of the International Association for the Study of Obesity*, *12*(5), e95–e106. https://doi.org/10.1111/j.1467-789X.2010.00769.x

Glanz, K., Bader, M. D. M., & Iyer, S. (2012). Retail grocery store marketing strategies and obesity: An integrative review. *American Journal of Preventive Medicine*. https://doi.org/10.1016/j.amepre.2012.01.013

Grasser, G., Van Dyck, D., Titze, S., & Stronegger, W. (2013). Objectively measured walkability and active transport and weight-related outcomes in adults: a systematic review. *International Journal of Public Health*, *58*(4), 615–625. https://doi.org/10.1007/s00038-012-0435-0

Hajna, S., Ross, N. A., Brazeau, A.-S., Belisle, P., Joseph, L., & Dasgupta, K. (2015). Associations between neighbourhood walkability and daily steps in adults: a systematic review and meta-analysis. *BMC Public Health*, *15*, 768. https://doi.org/10.1186/s12889-015-2082-x

Hollands, G. J., Shemilt, I., Marteau, T. M., Jebb, S. A., Kelly, M. P., Nakamura, R., … Ogilvie, D. (2013). Altering choice architecture to change population health behavior: a large-scale conceptual and empirical scoping review of interventions within micro-environments. *BMC Public Health*, *13*(December), 1–203. https://doi.org/10.1186/1471-2458-13-1218

Hunter, R. F., Christian, H., Veitch, J., Astell-Burt, T., Hipp, J. A., & Schipperijn, J. (2015). The impact of interventions to promote physical activity in urban green space: A systematic review and recommendations for future research. *Social Science & Medicine*, *124*, 246–256. https://doi.org/10.1016/j.socscimed.2014.11.051

Ikeda, E., Hinckson, E., Witten, K., & Smith, M. (2018). Associations of children's active school travel with perceptions of the physical environment and characteristics of the social environment: A systematic review. *Health & Place*, *54*, 118–131. https://doi.org/10.1016/j.healthplace.2018.09.009

Kaczynski, A. T., & Henderson, K. A. (2007). Environmental correlates of physical activity: A review of evidence about parks and recreation. *Leisure Sciences*. https://doi.org/10.1080/01490400701394865

Kahn-Marshall, J. L., & Gallant, M. P. (2012). Making Healthy Behaviors the Easy Choice for Employees. *Health Education & Behavior*, *39*(6), 752–776. https://doi.org/10.1177/1090198111434153

Karmeniemi, M., Lankila, T., Ikaheimo, T., Koivumaa-Honkanen, H., & Korpelainen, R. (2018). The Built Environment as a Determinant of Physical Activity: A Systematic Review of Longitudinal Studies and Natural Experiments. *Annals of Behavioral Medicine*, *52*(3), 239–251. https://doi.org/10.1093/abm/kax043

Kaushal, N., & Rhodes, R. E. (2014). The home physical environment and its relationship with physical activity and sedentary behavior: a systematic review. *Preventive Medicine*, *67*, 221–237. https://doi.org/10.1016/j.ypmed.2014.07.026

Kraak, V. I., Englund, T., Misyak, S., & Serrano, E. L. (2017). A novel marketing mix and choice architecture framework to nudge restaurant customers toward healthy food environments to reduce obesity in the United States. *Obesity Reviews*, *18*(8), 852–868. https://doi.org/10.1111/obr.12553

Krolner, R., Rasmussen, M., Brug, J., Klepp, K.-I., Wind, M., & Due, P. (2011). Determinants of fruit and vegetable consumption among children and adolescents: a review of the literature. Part II: qualitative studies. *The International Journal of Behavioral Nutrition and Physical Activity*, *8*, 112. https://doi.org/10.1186/1479-5868-8-112

Lachowycz, K., & Jones, A. P. (2011). Greenspace and obesity: A systematic review of the evidence. *Obesity Reviews*, *12*(501). https://doi.org/10.1111/j.1467-789X.2010.00827.x

Larson, N. I., Story, M. T., & Nelson, M. C. (2009). Neighborhood Environments. Disparities in Access to Healthy Foods in the U.S. *American Journal of Preventive Medicine*. https://doi.org/10.1016/j.amepre.2008.09.025

Lawman, H. G., & Wilson, D. K. (2012). A Review of Family and Environmental Correlates of Health Behaviors in High-Risk Youth. *Obesity*, *20*(6), 1142–1157. https://doi.org/10.1038/oby.2011.376

Lee, A. C. K., & Maheswaran, R. (2011). The health benefits of urban green spaces: a review of the evidence. *Journal of Public Health*, *33*(2), 212–222. https://doi.org/10.1093/pubmed/fdq068

Lee, L.-L., Kuo, Y.-L., & Chan, E. S.-Y. (2016). The Association Between Built Environment Attributes and Physical Activity in East Asian Adolescents: A Systematic Review. *Asia-Pacific Journal of Public Health*, *28*(3), 206–218. https://doi.org/10.1177/1010539516628174

Levy-Storms, L., Chen, L., & Loukaitou-Sideris, A. (2017). Older Adults' Needs and Preferences for Open Space and Physical Activity In and Near Parks: A Systematic Review. *Journal of Aging and Physical Activity*, 1–45. https://doi.org/10.1123/japa.2016-0354

Limstrand, T. (2008). Environmental characteristics relevant to young people's use of sports facilities: a review. *Scandinavian Journal of Medicine & Science in Sports*, *18*(3), 275–287. https://doi.org/10.1111/j.1600-0838.2007.00742.x

Luiu, C., Tight, M., & Burrow, M. (2018). Factors Preventing the Use of Alternative Transport Modes to the Car in Later Life. *Sustainability*, *10*(6). https://doi.org/10.3390/su10061982

Lovasi, G. S., Hutson, M. A., Guerra, M., & Neckerman, K. M. (2009). Built Environments and Obesity in Disadvantaged Populations. *Epidemiologic Reviews*, *31*(1), 7–20. https://doi.org/10.1093/epirev/mxp005

Lu, W., McKyer, E. L. J., Lee, C., Goodson, P., Ory, M. G., & Wang, S. (2014). Perceived barriers to children's active commuting to school: a systematic review of empirical, methodological and theoretical evidence. *The International Journal of Behavioral Nutrition and Physical Activity*, *11*, 140. https://doi.org/10.1186/s12966-014-0140-x

Maitland, C., Stratton, G., Foster, S., Braham, R., & Rosenberg, M. (2013). A place for play? The influence of the home physical environment on children's physical activity and sedentary behaviour. *International Journal of Behavioral Nutrition and Physical Activity*, *10*. https://doi.org/10.1186/1479-5868-10-99

Malambo, P., Kengne, A. P., De Villiers, A., Lambert, E. V, & Puoane, T. (2016). Built Environment, Selected Risk Factors and Major Cardiovascular Disease Outcomes: A Systematic Review. *PLoS ONE*, *11*(11). https://doi.org/10.1371/journal.pone.0166846

Marzi, I., Demetriou, Y., & Reimers, A. K. (2018). Social and physical environmental correlates of independent mobility in children:

a systematic review taking sex/gender differences into account. *International Journal of Health Geographics*, *17*. https://doi.org/10.1186/s12942-018-0145-9

Mayne, S. L., Auchincloss, A. H., & Michael, Y. L. (2015). Impact of policy and built environment changes on obesity-related outcomes: a systematic review of naturally occurring experiments. *Obesity Reviews : An Official Journal of the International Association for the Study of Obesity*, *16*(5), 362–375. https://doi.org/10.1111/obr.12269

McCormack, G. R., Rock, M., Toohey, A. M., & Hignell, D. (2010). Characteristics of urban parks associated with park use and physical activity: a review of qualitative research. *Health & Place*, *16*(4), 712–726. https://doi.org/10.1016/j.healthplace.2010.03.003

McCormack, G. R., & Shiell, A. (2011). In search of causality: a systematic review of the relationship between the built environment and physical activity among adults. *The International Journal of Behavioral Nutrition and Physical Activity*, *8*, 125. https://doi.org/10.1186/1479-5868-8-125

McCrorie, P. R. W., Fenton, C., & Ellaway, A. (2014). Combining GPS, GIS, and accelerometry to explore the physical activity and environment relationship in children and young people - a review. *The International Journal of Behavioral Nutrition and Physical Activity*, *11*, 93. https://doi.org/10.1186/s12966-014-0093-0

McGrath, L. J., Hopkins, W. G., & Hinckson, E. A. (2015). Associations of objectively measured built-environment attributes with youth moderate-vigorous physical activity: a systematic review and meta-analysis. *Sports Medicine (Auckland, N.Z.)*, *45*(6), 841–865. https://doi.org/10.1007/s40279-015-0301-3

MacMillan, F., George, E. S., Feng, X., Merom, D., Bennie, A., Cook, A., … Astell-Burt, T. (2018). Do Natural Experiments of Changes in Neighborhood Built Environment Impact Physical Activity and Diet? A Systematic Review. *International Journal of Environmental Research and Public Health*, *15*(2).

Mhurchu, C. N., Vandevijvere, S., Waterlander, W., Thornton, L. E., Kelly, B., Cameron, A. J., … INFORMAS. (2013). Monitoring the availability of healthy and unhealthy foods and non-alcoholic beverages in community and consumer retail food environments globally. *Obesity reviews*, *14*(1, SI), 108–119. https://doi.org/10.1111/obr.12080

Moran, M., Van Cauwenberg, J., Hercky-Linnewiel, R., Cerin, E., Deforche, B., & Plaut, P. (2014). Understanding the relationships

between the physical environment and physical activity in older adults: a systematic review of qualitative studies. *The International Journal of Behavioral Nutrition and Physical Activity*, *11*, 79. https://doi.org/10.1186/1479-5868-11-79

Moschonis, G., Tsoutsoulopoulou, K., Efstathopoulou, E., Tsirigoti, L., Lambrinou, C.-P., Georgiou, A., … Manios, Y. (2015). Conceptual framework of a simplified multi-dimensional model presenting the environmental and personal determinants of cardiometabolic risk behaviors in childhood. *Expert Review of Cardiovascular Therapy*, *13*(6), 673–692. https://doi.org/10.1586/14779072.2015.1039992

Nocon, M., Muller-Riemenschneider, F., Nitzschke, K., & Willich, S. N. (2010). Review Article: Increasing physical activity with point-of-choice prompts--a systematic review. *Scandinavian Journal of Public Health*, *38*(6), 633–638. https://doi.org/10.1177/1403494810375865

Oliveira, A. F., Moreira, C., Abreu, S., Mota, J., & Santos, R. (2014). Environmental determinants of physical activity in children: A systematic review. *Archives of Exercise in Health and Disease*, *4*(2), 254–261. https://doi.org/10.5628/aehd.v4i2.158

Panter, J. R., Jones, A. P., & van Sluijs, E. M. (2008). Environmental determinants of active travel in youth: a review and framework for future research. *The International Journal of Behavioral Nutrition and Physical Activity*, *5*, 34. https://doi.org/10.1186/1479-5868-5-34

Parrish, A. M., Okely, A. D., Stanley, R. M., & Ridgers, N. D. (2013). The effect of school recess interventions on physical activity: A systematic review. *Sports Medicine*. https://doi.org/10.1007/s40279-013-0024-2

Pont, K., Ziviani, J., Wadley, D., Bennett, S., & Abbott, R. (2009). Environmental correlates of children's active transportation: A systematic literature review. *Health and Place*, *15*(3), 827–840. https://doi.org/10.1016/j.healthplace.2009.02.002

Pucher, J., Dill, J., & Handy, S. (2010). Infrastructure, programs, and policies to increase bicycling: An international review. *Preventive Medicine*, *50*(1), S106–S125. https://doi.org/10.1016/j.ypmed.2009.07.028

Rahmanian, E., Gasevic, D., Vukmirovich, I., & Lear, S. A. (2014). The association between the built environment and dietary intake - a systematic review. *Asia Pacific Journal of Clinical Nutrition*, *23*(2), 183–196.

Renalds, A., Smith, T. H., & Hale, P. J. (2010). A systematic review of built environment and health. *Family & Community Health*, *33*(1), 68–78. https://doi.org/10.1097/FCH.0b013e3181c4e2e5

Rhodes, R. E., Saelens, B. E., & Sauvage-Mar, C. (2018). Understanding Physical Activity through Interactions Between the Built Environment and Social Cognition: A Systematic Review. *Sports Medicine*, *48*(8), 1893–1912. https://doi.org/10.1007/s40279-018-0934-0

Ridgers, N. D., Salmon, J., Parrish, A.-M., Stanley, R. M., & Okely, A. D. (2012). Physical activity during school recess: a systematic review. *American Journal of Preventive Medicine*, *43*(3), 320–328. https://doi.org/10.1016/j.amepre.2012.05.019

Rosso, A. L., Auchincloss, A. H., & Michael, Y. L. (2011). The urban built environment and mobility in older adults: A comprehensive review. *Journal of Aging Research*, *2011*. https://doi.org/10.4061/2011/816106

Rothman, L., Macpherson, A. K., Ross, T., & Buliung, R. N. (2018). The decline in active school transportation (AST): A systematic review of the factors related to AST and changes in school transport over time in North America. *Preventive Medicine*, *111*, 314–322. https://doi.org/10.1016/j.ypmed.2017.11.018

Saelens, B. E., & Handy, S. L. (2008). Built environment correlates of walking: A review. *Medicine and Science in Sports and Exercise*. https://doi.org/10.1249/MSS.0b013e31817c67a4

Salvo, G., Lashewicz, B. M., Doyle-Baker, P. K., & McCormack, G. R. (2018). Neighbourhood Built Environment Influences on Physical Activity among Adults: A Systematized Review of Qualitative Evidence. *International Journal of Environmental Research and Public Health*, *15*(5). https://doi.org/10.3390/ijerph15050897

Sawyer, A., Ucci, M., Jones, R., Smith, L., & Fisher, A. (2017). Simultaneous evaluation of physical and social environmental correlates of physical activity in adults: A systematic review. *SSM - Population Health*, *3*, 506–515. https://doi.org/10.1016/j.ssmph.2017.05.008

Schüle, S. A., & Bolte, G. (2015). Interactive and independent associations between the socioeconomic and objective built environment on the neighbourhood level and individual health: A systematic review of multilevel studies. *PLoS ONE*, *10*(4). https://doi.org/10.1371/journal.pone.0123456

Schulz, M., Romppel, M., & Grande, G. (2018). Built environment and health: a systematic review of studies in Germany. *Journal of Public Health*, *40*(1), 8–15. https://doi.org/10.1093/pubmed/fdw141

Sharmin, S., & Kamruzzaman, M. (2017). Association between the built environment and children's independent mobility: A meta-analytic review. *Journal of Transport Geography*, *61*, 104–117. https://doi.org/10.1016/j.jtrangeo.2017.04.004

Shields, N., Synnot, A. J., & Barr, M. (2012). Perceived barriers and facilitators to physical activity for children with disability: a systematic review. *British Journal of Sports Medicine*. https://doi.org/10.1136/bjsports-2011-090236

Sirard, J. R., & Slater, M. E. (2008). Walking and Bicycling to School: A Review. *American Journal of Lifestyle Medicine*. https://doi.org/10.1177/1559827608320127

Smith, M., Hosking, J., Woodward, A., Witten, K., MacMillan, A., Field, A., … Mackie, H. (2017). Systematic literature review of built environment effects on physical activity and active transport - an update and new findings on health equity. *International Journal of Behavioral Nutrition and Physical Activity*, *14*. https://doi.org/10.1186/s12966-017-0613-9

Soler, R. E., Leeks, K. D., Buchanan, L. R., Brownson, R. C., Heath, G. W., & Hopkins, D. H. (2010). Point-of-decision prompts to increase stair use. A systematic review update. *American Journal of Preventive Medicine*, *38*(2 Suppl), S292-300. https://doi.org/10.1016/j.amepre.2009.10.028

Stanley, R. M., Ridley, K., & Dollman, J. (2012). Correlates of children's time-specific physical activity: A review of the literature. *International Journal of Behavioral Nutrition and Physical Activity*. https://doi.org/10.1186/1479-5868-9-50

Stappers, N. E. H., Van Kann, D. H. H., Ettema, D., De Vries, N. K., & Kremers, S. P. J. (2018). The effect of infrastructural changes in the built environment on physical activity, active transportation and sedentary behavior - A systematic review. *Health & Place*, *53*, 135–149. https://doi.org/10.1016/j.healthplace.2018.08.002

Starnes, H. A., Troped, P. J., Klenosky, D. B., & Doehring, A. M. (2011). Trails and Physical Activity: A Review. *Journal of Physical Activity & Health*, *8*(8), 1160–1174. https://doi.org/10.1123/jpah.8.8.1160

Stewart, O. (2011). Findings from research on active transportation to school and implications for safe routes to school programs. *Journal of Planning Literature*, *26*(2), 127–150. https://doi.org/10.1177/0885412210385911

Sugiyama, T., Neuhaus, M., Cole, R., Giles-Corti, B., & Owen, N. (2012). Destination and Route Attributes Associated with Adults' Walking: A Review. *Medicine and Science in Sports and Exercise*, *44*(7), 1275–1286. https://doi.org/10.1249/MSS.0b013e318247d286

Van Cauwenberg, J., De Bourdeaudhuij, I., De Meester, F., Van Dyck, D., Salmon, J., Clarys, P., & Deforche, B. (2011). Relationship between the physical environment and physical activity in older adults: A systematic review. *Health and Place*, *17*(2), 458–469. https://doi.org/10.1016/j.healthplace.2010.11.010

Van Cauwenberg, J., Nathan, A., Barnett, A., Barnett, D. W., Cerin, E., CEPA, & Grp, O. A. W. (2018). Relationships Between Neighbourhood Physical Environmental Attributes and Older Adults' Leisure-Time Physical Activity: A Systematic Review and Meta-Analysis. *Sports Medicine*, *48*(7), 1635–1660. https://doi.org/10.1007/s40279-018-0917-1

Van Hecke, L., Ghekiere, A., Veitch, J., Van Dyck, D., Van Cauwenberg, J., Clarys, P., & Deforche, B. (2018). Public open space characteristics influencing adolescents' use and physical activity: A systematic literature review of qualitative and quantitative studies. *Health and Place*, *51*, 158–173. https://doi.org/10.1016/j.healthplace.2018.03.008 LK

Van Holle, V., Deforche, B., Van Cauwenberg, J., Goubert, L., Maes, L., de Weghe, N., & De Bourdeaudhuij, I. (2012). Relationship between the physical environment and different domains of physical activity in European adults: a systematic review. *BMC Public Health*, *12*. https://doi.org/10.1186/1471-2458-12-807

Wendel-Vos, W., Droomers, M., Kremers, S., Brug, J., & van Lenthe, F. (2007). Potential environmental determinants of physical activity in adults: a systematic review. *Obesity Reviews : An Official Journal of the International Association for the Study of Obesity*, *8*(5), 425–440. https://doi.org/10.1111/j.1467-789X.2007.00370.x

Williams, J., Scarborough, P., Matthews, A., Cowburn, G., Foster, C., Roberts, N., & Rayner, M. (2014). A systematic review of the influence of the retail food environment around schools on obesity-related outcomes. *Obesity Reviews*, *15*(5), 359–374. https://doi.org/10.1111/obr.12142

Wong, B. Y.-M., Faulkner, G., & Buliung, R. (2011). GIS measured environmental correlates of active school transport: a systematic review of 14 studies. *The International Journal of Behavioral Nutrition and Physical Activity*, *8*, 39. https://doi.org/10.1186/1479-5868-8-39

Yen, I. H., Fandel Flood, J., Thompson, H., Anderson, L. A., & Wong, G. (2014). How design of places promotes or inhibits mobility of older adults: realist synthesis of 20 years of research. *Journal of Aging and Health*, *26*(8), 1340–1372. https://doi.org/10.1177/0898264314527610

Zapata-Diomedi, B., & Veerman, J. L. (2016). The association between built environment features and physical activity in the Australian context: a synthesis of the literature. *BMC Public Health*, *16*, 484. https://doi.org/10.1186/s12889-016-3154-2

Zhang, R., Wulff, H., Duan, Y., & Wagner, P. (2019). Associations between the physical environment and park-based physical

activity: A systematic review. *Journal of Sport and Health Science*. https://doi.org/10.1016/j.jshs.2018.11.002 LK
